# Health vulnerabilities of readymade garment (RMG) workers: a systematic review

**DOI:** 10.1186/s12889-019-6388-y

**Published:** 2019-01-15

**Authors:** Humayun Kabir, Myfanwy Maple, Kim Usher, Md Shahidul Islam

**Affiliations:** 1 School of Health, Faculty of Medicine and Health, University of New England, Armidale, NSW 2351 Australia; 2 Department of Sociology, University of Dhaka, Dhaka, 1000 Bangladesh

**Keywords:** Readymade garment, Health vulnerability, South and Southeast Asia, Systematic review

## Abstract

**Background:**

There is a paucity of literature that addresses the health vulnerabilities of readymade garment (RMG) workers in South and Southeast Asian regions. Therefore, the aim of this systematic review is to identify the distinctive types of health vulnerabilities along with the causes and consequences of these vulnerabilities of the RMG workers in South and Southeast Asian regions.

**Methods:**

Systematic review search methods were applied utilising the PRISMA protocol. Literature published between July 2007 to June 2017 on health vulnerabilities of the RMG workers of South and Southeast Asian countries were identified through electronic databases and manual searches.

**Results:**

A total number of 19 studies (16 quantitative studies, 3 mixed-method studies) were included from the primary 17,001 papers identified. The quality of these studies was assessed by using the EPHPP (effective public health practice project) and the CASP (critical appraisal skills programme) tools. From the identified studies, 14 were considered ‘strong,’ with the remainder assessed as ‘moderate’ quality. The findings reported in these studies suggest that RMG workers of South and Southeast Asian countries are prone to several health vulnerabilities which include physical and psychological issues. Further, many of these health vulnerabilities arise from the nature of the RMG workplace, and include unhygienic and unsafe working environments, hazardous conditions of the factories, and lack of safety equipment.

**Conclusions:**

This systematic review suggests that RMG workers’ health vulnerabilities are an emerging area of inquiry that needs to be better understood and solutions identified. Little is currently known about the distinctive types of health vulnerabilities of the RMG workers of these countries, other than Bangladesh and India, due to the lack of robust studies in other South and Southeast Asian countries. Although the health vulnerabilities of the Bangladeshi and Indian RMG workers have been previously highlighted, the health vulnerabilities arising from sudden disasters in the sector remain a neglected issue.

## Background

### South and Southeast Asian focus on readymade garment (RMG) workers’ health vulnerabilities

Garment workers globally experience distinctive vulnerabilities in the workplace [1, 2]. These occupational vulnerabilities are related to dangerous and unhealthy working conditions, and employment in sub-standard physical environments which can result in fires and full or partial collapse of buildings. In addition, sexual harassment at workplace, low wages and repetitive strain from physically demanding and intense work also make the workers vulnerable. However, health issues represent the most significant among the vulnerabilities faced by the workers during their working tenure in the readymade garment (RMG) sector [3–5]. While the extent and nature of health vulnerabilities related to working in the RMG trade vary between countries, it is evident that RMG workers of South and Southeast Asia are the most affected by the unhygienic and unsafe nature of their workplace conditions [3, 4, 6–10]. Specific countries of South and Southeast Asia (such as China, Bangladesh, India, Thailand, Vietnam, Cambodia, Philippines, Sri Lanka, and Pakistan) can be considered the main RMG product exporters to the world mainly because of cheap (i.e. low wages) and available labour, simple or no technology/prior skills required for the job, and the lack of alternative job options, particularly for women [3, 11–13]. While low-cost employment availability is important, factory owners tend to minimize production costs of RMG merchandise so that they can be competitive and attract international buyers. As a result, less money is invested in providing a secure working environment for the RMG factories which eventually makes the health of RMG workers vulnerable [3].

This systematic review focuses on understanding workplace conditions and the impact of these conditions on RMG workers’ physical and psychological health. While South and Southeast Asia house the majority of RMG factories, no prior reviews have been located that seek to understand the health vulnerabilities of these workers. Consequently, this systematic review will provide an overview of the available evidence related to the physical and psychological health vulnerabilities of RMG workers in South and Southeast Asian countries.

### Importance of studying RMG workers’ health vulnerabilities

RMG workers are frequently affected by various types of diseases mainly due to the unhygienic workplace conditions and the dust produced from raw materials [6, 7]. They are reported as developing several health issues including coughs, fevers, jaundice, kidney failure, musculoskeletal problems, respiratory problems, and sexually transmitted diseases such as HIV/AIDS [4, 14–17] as a result of their employment, and thus RMG workers experience illness and become unable to continue working. Consequently, they are less productive and may lose their employment. Furthermore, as most RMG workers do not get full payment when on sick leave, concerns about failing health may lead to stress and psychological health issues, in addition to the financial burden. Similar to employees in other sectors, RMG workers have the right to work in an environment which is safe and without risks to their health. The International Labor Organization (ILO) convention 155 (1981) on Occupational Safety and Health also determines the acts to be taken by the employers to provide and maintain safe and healthy working conditions [18]. Ironically, better health conditions for the RMG workers would benefit the factory owners and the economy, in the long term. For example, if workers do not get sick then they will be able to continue to work all the year round which would increase RMG productions [19]. Therefore, it is important to know the underlying causes and consequences of health vulnerabilities for RMG workers, as understanding these issues will help to explore an effective way to ensure conditions in the workplace do not expose the workers to these vulnerabilities in the future.

### Gap in the literature

There is a dearth of literature focusing specifically on both the physical and psychological health vulnerabilities of RMG workers in South and Southeast Asian countries. Therefore, this systematic review aims to identify what is currently known about the health vulnerabilities of RMG workers in South and Southeast Asian countries. The question driving this research is: “What are the health vulnerabilities of RMG workers in South and Southeast Asia?” This research question is addressed through a systematic review of the existing evidence. In this paper, we seek to identify distinctive types of health vulnerabilities, along with the causes and consequences of these health vulnerabilities.

## Methods

### Search strategy

This review was undertaken using the process outlined in the PRISMA (Preferred Reporting Items for Systematic Reviews and Meta-Analyses) guidelines [20, 21]. Relevant studies for this systematic review were identified by screening four electronic databases (PubMed Central, ProQuest full text, CINAHL Complete, and Google Scholar). It is worth noting here that although Google Scholar is a non-scientific search engine, it was included to ensure we captured all available evidence from the South and Southeast Asian regions which may have been published in journals not indexed on the included databases. Searching for relevant published literature was undertaken from May to September 2017 and restricted to the research articles published over a ten-year period from July 2007 to June 2017 to identify the contemporary health vulnerabilities, along with the causes of these vulnerabilities, among RMG workers from South and Southeast Asian countries.

**Fig. 1:**
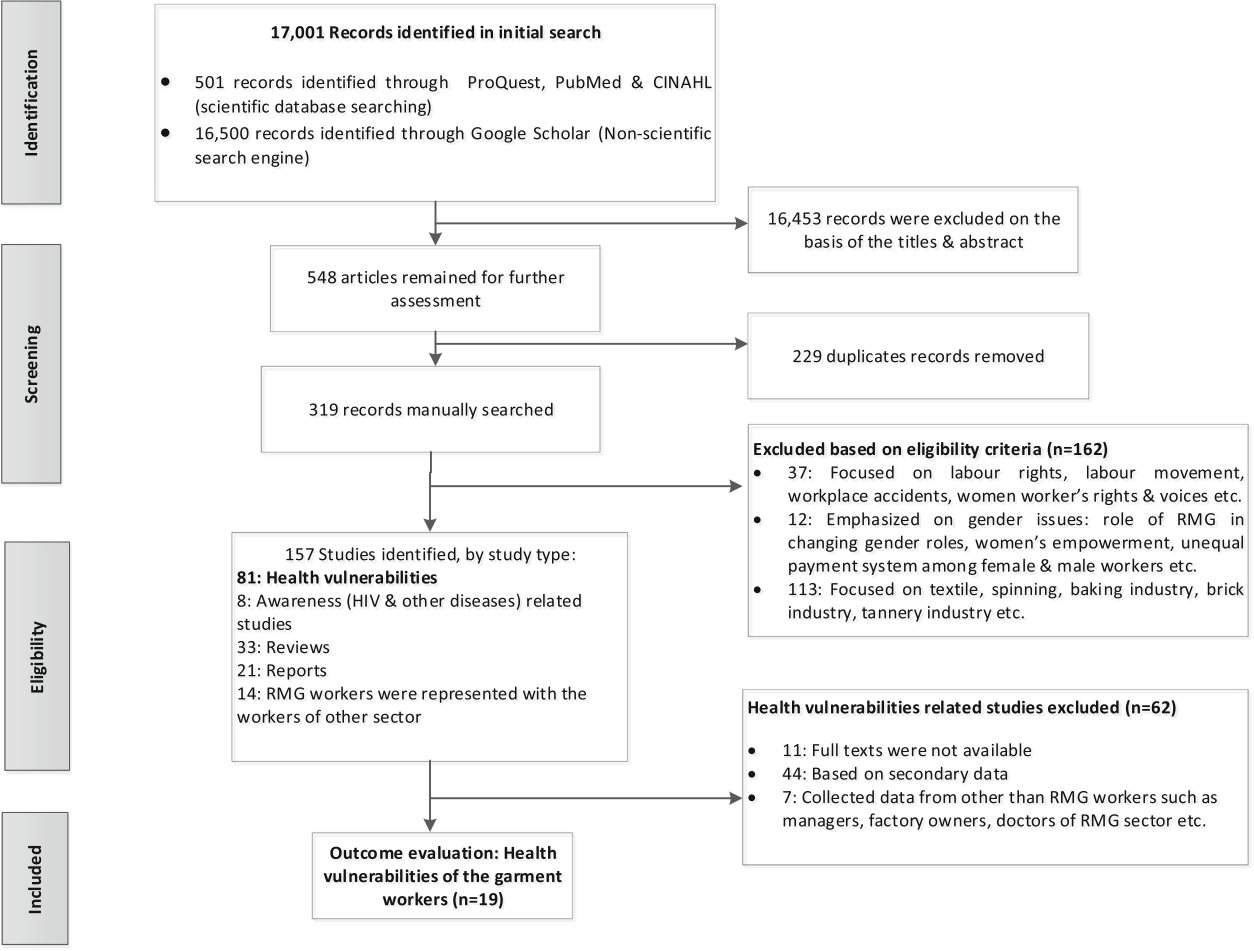
PRISMA flowchart

The keywords that were used in the search were combinations of: 1) health problems OR health risks OR heath vulnerabilities OR health status; 2) by the name of the particular country (e.g. Bangladesh, India, Nepal, Bhutan, Pakistan, Maldives, Sri Lanka, Afghanistan) of South Asia and Southeast Asia (e.g. Philippines, Indonesia, Malaysia, Thailand, China, Cambodia, Myanmar, Laos, Singapore, Vietnam, Brunei, Timor-Leste); and 3) readymade garment OR garment factory/industry OR clothing industry/factory as presented in the PRISMA flow chart (Fig. 1). Initially, these keywords were searched in the title and abstract of papers found through using the aforementioned search engines. In addition, the reference lists of the potential papers/evidence were also searched to identify any further potential evidence. The search strategy, including determination of keywords of the search items in the title and abstract of the articles, was fixed by the author (HK) in consultation with the librarian of the University of New England and agreed by all authors.

### Eligibility criteria

#### Inclusion criteria

- Peer-reviewed empirical research published between July 2007 and June 2017;
- Article included data on health vulnerability and/or causes of vulnerability;
- Published in English;
- Data collected from RMG workers in Southeast and/or South Asian Counties.

#### Exclusion criteria

- Research related to other than health vulnerabilities issues (such as awareness about hygienic practices, labor rights movement, paid work and socio-political consciousness, workplace conditions, gender issues) of the RMG workers of the South and Southeast Asian countries were excluded;
- Research articles that focused on cotton mill/industry, textile mill, and silk industry in particular, were also excluded;
- Literature based on secondary data and review articles were not considered for the review.

## Results

### Search results

The primary searches included 17,001 articles and after excluding 16,453 records (on the basis of abstracts and titles) 548 articles remained. 229 duplicates were removed, with 319 studies remaining for further consideration. From the manually searched items (*n* = 319), 162 studies were excluded (on the basis of whole articles) after applying the eligibility criteria. As a result, 157 records were identified by study type of which 81 articles focused on health vulnerabilities of the RMG workers in the countries specified. Of these 81 articles, 62 studies were removed because of unavailability of full texts (*n* = 11), written based on secondary data (*n* = 44), and where RMG workers were not the primary respondents (*n* = 7). From this process, nineteen articles (16 quantitative, 3 mixed-methods) were included in the systematic review (Fig. 1 PRISMA flow chart). No qualitative studies were identified. Articles that were finally included focused on RMG workers in Bangladesh (*n* = 10), India (*n* = 4), Cambodia (n = 1), Sri Lanka (*n* = 2), China (n = 1), and Thailand (n = 1). Women constituted almost two-thirds of the study populations presented in the studies. The majority of the studies (n = 10) included both male and female workers to collect the data and eight papers focused solely on female workers as the study respondents. One article did not specify gender in the sample [22].

### Overview of the studies

#### Health vulnerabilities among the garment workers: evidence from quantitative studies

The majority of the articles (*n* = 10) were identified as cross-sectional studies and included participants for face to face interview using structured or semi-structured questionnaires. Along with these face to face interviews, five studies examined blood samples, hair, skin test, heights and weights of the respondents to understand health vulnerabilities related symptoms (such as stress level, nutritional status, morbidity pattern, cardiovascular risk factors, and skin diseases) of the participants. From the results, RMG workers’ health vulnerabilities are categorized into physical and psychological health vulnerabilities (see Table 1).

**Table 1:** Research on health vulnerabilities in the RMG sector: based on quantitative methods

Author(s) & Year of publication (Country)	Title	Methodology	Theme/focusing point	Findings/outcomes	Limitations	Quality grading
Padmini & Venmathi, 2012 [22] (India)	Unsafe work environment in garment industries, Tirupur, India	Quantitative. Sample size: 514. Respondents (participated in a face to face confidential interview) were included from 13 small, medium & large scale garment industries for this study.	To measure correlations between workplace safety issues (i.e. hazards) & health status of the respondents.	The percentage of experiencing different kinds of hazards (by the RMG workers) which have negative impacts on workers’ health status are given below: (a) Ergonomic hazards (e.g. inadequate seating & standing arrangements for the workers, obsolete machinery, improper lifting or movement of heavy loads): 67.5%. (b) Physical hazards (e.g. noise, lighting problem, electric shock, hot conditions inside the factory, ventilation problems etc.): 34.6%. (c) Psychological hazards (e.g. monotonous type of work, feeling of risks at workplace, long working hours, lack of recognition etc.): 32.6%. (d) Mechanical hazards (e.g. fire hazard): 25%. (e) Chemical hazards (e.g. dust, fumes, mist, smoke etc.): 11.6%.	Respondents were not identified clearly (whether the study respondents were male or female were not clearly mentioned). Sampling procedure is not clear.	Moderate
Khan et al., 2015 [24] (Bangladesh)	Occupational health hazards among workers of garment factories in Dhaka city, Bangladesh	Cross-sectional study. Quantitative. Sample size: 145. Female: 89%. Male: 11% Study location: Dhaka. Purposive sampling procedure was followed. Structured questionnaire was used to collect the data through face to face interview. Data were analyzed using SPSS 17.	To focus on how physical settings (i.e. dirty, inadequate light, noise pollution, overcrowding, problem with safe drinking water, etc.) of factories create health hazards (e.g., headache or shoulder pain, backache, joint pain, eye strain, hearing problem, gastroenteritis, chest pain, breathing difficulty, skin disease, tuberculosis, insomnia etc.) among the respondents.	Prevalence of occupational health hazards among the respondents: 88.28%. Symptoms of health vulnerabilities: Headache (51%), Joint pain (31%), General weakness (28.3%), Chest pain (26.2%), Backache (24.8%), Gastroenteritis (21.4%), Jaundice (20.7%), Insomnia (20%), Eye strain/problem (13.8%), Hearing problem (8.3%), Skin disease (5.5%), Breathing problem (3.4%), Tuberculosis (2.8%). Causes identified: Bad physical environment; such as noise pollution (33.8%), problem with safe drinking water (15.9%), overcrowding (13.8%), inadequate light (9.7%), dirty (9%), unavailability of separate toilets (5.5%), inadequate ventilation (4.1%); & dusty raw materials of the factories.	Psychological health issues are ignored while psychological health is also similarly important such as physical health.	Moderate
Chumchai et al., 2015 [7] (Thailand)	Prevalence and risk factors of respiratory symptoms among home-based garment workers in Bangkok, Thailand	Cross-sectional study. Quantitative. Sample size: 300. Male: 66 (22%). Female: 232 (78%). Respondents were selected randomly. Used SPSS for data analysis. Logistic regression analysis was applied to identify risk factors associated with respiratory symptoms.	To determine the prevalence and risk factors related to respiratory symptoms.	Prevalence of respiratory problems among the respondents is: 22.3%. Common symptoms of respiratory problems: Abnormal lung function (29.3%) Nasal congestion (17.3%) Cough (5%) Itchiness (4.7%) Phlegm (4%) Cough with sputum (1.7%) Chest tightness (0.03%). Allergic symptoms: 25.3%. Causes of respiratory problems/factors associated with respiratory symptoms: Allergic reaction (61.8%) & Fabric dust (61.5%).	Ambiguity about sample size: the abstract and methodology say that the size of respondents is 300, however, Table 1 shows that the sample size is 298 (male: 232; female: 66). RMG factory workers were not included, which could give a broader perspective on the existing respiratory problems of the workers working in the factories.	Strong
Steinisch et al., 2014 [31] (Bangladesh)	Work stress and hair cortisol levels among workers in a Bangladeshi ready-made garment factory-results from a across-sectional study	Cross-sectional interview-based study. Quantitative. Sample size: 175. Female: 131. Male: 44. Study location: Dhaka. Hair Cortisol Concentrations (HCC) were analyzed by liquid chromatography-mass spectrometry.	To explore associations between work stress (work-related demands, interpersonal resources, & work-related values) & long-term integrated cortisol levels in hair among the respondents.	Causes of HCC (Hair Cortisol Concentrations): Work-related values: (a) Lack of job security, & (b) Lack of promotion prospects Lack of job promotion prospect may cause poorer mental health because it is a rare case in RMG sector and requires exceptional job related demands (i.e. promotion prospects are strongly associated with high work stress).	Only 34% of the respondents, who were interviewed, gave their hair samples. Therefore, the HCC test is questionable.	Strong
Ahmed & Raihan, 2014 [4] (Bangladesh)	Health status of the female workers in the garment sector of Bangladesh	Quantitative. Sample size: 200 (female). Respondents were selected from 15 leading garment factories. Study location: Gazipur, Savar (Dhaka). Respondents were interviewed using structured questionnaire. Data were analyzed using SPSS (both descriptive & inferential analyses were done with survey data).	To show respondents’ experiences of major diseases due to working in the RMG sector of Bangladesh.	15 diseases were identified that are mainly responsible for health vulnerabilities of the respondents: Fever (81.5%), Common cold (79%), Abdomen pain (75.5%), Fatigue (75%), Gastric pain (71.5%), Back pain (68%), Malnutrition (65.5%), Pruritus (59%), Helminthiasis (58%), Dermatitis (57%), Problems in bones (57%), Eye stain/problem (56.5%), Hepatitis (Jaundice) (51.5%), Respiratory problems (46%), Abortion due to retain job (35.5%). Psychological problem: Trauma (52%). Causes: unhealthy environment, long working hours, imbalanced diet, & sexual contact.	Issues on psychological health were not focused broadly & male workers were not included in the study	Strong
Shanbhag & Bobby, 2012 [8] (India)	Mental health status of female workers in private apparel manufacturing industry in Bangalore city, Karnataka, India	Descriptive study. Quantitative. Sample size: 350 (female). Respondents were selected randomly from 3 units of a private garment factory. Chi-square test was done.	To assess factors affecting the mental health status of the respondents.	Prevalence of mental illness among the respondents: 39% Symptoms: Somatic illness (11%) Anxiety (7.6%) Social dysfunction (7.1%) Depression (6.8%).	The causes of mental health problems were not explored. Male workers were excluded.	Strong
Chen et al., 2017 [6] (China)	Survey of occupational allergic contact dermatitis and patch test among clothing employees in Beijing	Cross-sectional study. Quantitative. Sample size: 529. Male: 299. Female: 230. Respondents were selected from 12 clothing factories through using quota sampling procedure. Self-administered questionnaire was used for face to face interviewing. Skin of all respondents were tested by a dermatologist.	To investigate the prevalence of occupational allergic contact dermatitis and causes of allergy among the respondents.	Overall 1 year prevalence of occupational allergic contact dermatitis (OACD) among the clothing employees: 8.5%. (a) Prevalence among the workers: 10.8% (b) Prevalence among managers: 3.2% Locations of skin complaints: Hand/wrists, Forearms, Face/neck, & Trunk. Causes: Work materials. Note: OACD is less prevalent among the managers compared to the workers.	Sampling procedure is not well explained.	Strong
Steinisch et al., 2013 [10] (Bangladesh)	Work stress: its components and its association with self-reported health outcomes in a garment factory in Bangladesh-Findings from a cross-sectional study	Cross-sectional epidemiological study. Quantitative. Sample size: 332. Male: 54. Female: 278. Study location: Dhaka.	To identify the causes & consequences of work stress among the respondents.	Self-reported poor health: 41%. Symptoms: Cold (51.8%), Headache (48.2%), Back pain (26.2%), Muscle cramps (26%), Sleeplessness (22.3%), Stomach problem (16.3%), Breathing problem (13%), Jaundice (6%). Components/causes of work stress: (a) Work related demands: Physical demand (62%), Time pressure (59.6%), Worries about making mistakes (62.3%), Exposure to abusive language (33.4%) (b) Work related values: Lack of freedom at work (43.1%), Lack of promotion prospects (42.2%), & Lack of job security (38.9%).	Workers’ feelings of stress/risk, which can be produced from a sudden disaster such as collapse of factory building & fire in the factory building, was not considered in this research.	Strong
Fatema et al., 2014 [29] (Bangladesh)	Cardiovascular risk factors among Bangladeshi ready-made garment workers	Quantitative. Sample size: 614. Male: 313. Female: 301. Respondents were recruited from 6 garments following simple random sampling. (Epidemiological study conducted through screening of the workers in the medical service centre of EPZ area).	To estimate the prevalence & identifying the correlation between anthropometry & the clinical risk factor for cardiovascular diseases (CVDs) among the respondents.	80.6% of the respondents had at least one of the CVDs risk factors. Prevalence of CVDs risk factors: Obesity (27.9%), Overweight (23%), Triglyceride (19.7%), Hypertension (14.5%), Cholesterol (9.1%), Diabetes (7.3%). • Male workers are vulnerable to hypertension due to excessive smoking habit. • Female workers are at risks to diabetes due to over-weight & central obesity.	The causes of CVDs were not addressed in this study.	Moderate
Makurat et al., 2016 [11] (Cambodia)	Nutritional and micronutrient status of female workers in a garment factory in Cambodia	Cross-sectional study. Quantitative. Sample size: 223 (female only). Used semi-structured questionnaire. Blood samples of the respondents were tested to obtain nutritional and micronutrient status. Bivariate analysis was done.	To examine nutritional, hemoglobin as well as the micronutrient status of the respondents.	Symptoms of health risks: Marginal iron store (46.5%) Underweight (31.4%) Anemia (26.9%) Iron deficiency (22.1%). Self-reported sickness for which respondents took 14 days sick leave: Respiratory tract infection (45.7%) Fever (30.9%) Diarrhea (20.2%). The cause of these diseases: Poor nutritional status. The cause of poor nutritional status: Minimum salaries.	Exclusion of male respondents is not justified.	Strong
Fitch et al., 2017 [25] (Bangladesh)	Prevalence and risk factors of depression among garment workers in Bangladesh	Quantitative. Sample size: 308 (female only). Study location: Dhaka. Respondents were purposively chosen for the survey. Snowball sampling procedure was applied. Data were analyzed using univariate & multivariate analysis.	To explore the incidence of depression and its related risk factors among the female garment workers.	Prevalence of depression (moderate to severe) among the garment workers: 20.9%. Risk factors associated with depression (moderate to severe): Joint pain (44.1%), Anxiety (43.8%), Vision/eye problems (41.9%), Dysuria (41.7%), Insomnia (39.5%), Gout (39.3%), Hypertension (33.3%), Diabetes (31.6%), Asthma (28.6%), Heart attack (25%). Causes: Work related risk factors such as pat-time work, low income, job insecurity, unhealthy workplace conditions.	Number of factories, from where data were collected, were not mentioned clearly	Strong
Hasnain et al., 2014 [26] (Bangladesh)	Morbidity patterns, nutritional status, and healthcare-seeking behavior of female garment workers in Bangladesh	Cross-sectional study. Quantitative. Sample size: 300 (female only). Respondents were purposively selected for interviewing using semi-structured questionnaire from 2 factories. Study location: Dhaka. Respondents’ heights & weights were measured according to the guideline of WHO. The Chi-square test was used & data were analyzed by SPSS 16.01.	To determine female garment workers’ nutritional status, their different types of health-related problems, & healthcare-seeking behavior.	Prevalence of different kinds of health problems: 53.67%. Symptoms & signs: Underweight (43.33%), Anemia (31%), Anorexia (22.33%), Nausea (14.33%), Fever (11.67%), Epigastric pain (11.33%), Dysmenorrhea (10.33%), Burning micturition (10.67%), Lower abdominal pain (7%), Headache (7.67%), Cough (8%), Runny nose (5.67%), Diarrhea (4.33%), Vertigo (4.67%), Vomiting (4%), Menorrhagia (3%), Leg pain (2.33%). Causes of more vulnerable to illness: Poor economic status & low educational levels. • 96% of the underweight respondents had one or more health-related problems in the last three months. • Only 11.67% of the respondents go to receive healthcare services from the qualified private & government doctors.	Questions regarding malnutrition were not included in the semi-structured interview questionnaire. Male workers were excluded from the study.	Strong
Parimalam et al., 2007 [27] (India)	Knowledge, attitude, practices related to occupational health problems among garment workers in Tamil Nadu, India.	Cross sectional study. Quantitative. Sample size: 216. Male: 91 (42%). Female: 125 (58%). Respondents were selected by following stratified random sampling from 3 different sections (cutting, stitching, & finishing sections) of the garment industry for face to face confidential interview. The Chi-square test was used to analyze the data.	To understand the common health problems of the garment workers & to assess their level of awareness about these problems.	Major types of health problems faced by garment workers from different sections: (a) Neural: Cutting (18.5%), Stitching (84.7%), Finishing (25%). (b) Hearing disability: Cutting (11.1%), Stitching (34.4%), Finishing (6.9%). (c) Dermatological: Cutting (11.1%), Stitching (9.9%), Finishing (6.9%). (d) Respiratory: Cutting (84%), Stitching (21.4%), Finishing (10.3%). (e) Musculoskeletal discomforts: Cutting (33.3%), Stitching (83.2%), Finishing (34.5%). Causes of these health problems: Work environment, workstation design, constrained work posture, lack of safety related training & safety tools, & other occupational risk factors such as force, task duration, frequency or repetitiveness of movement etc.	Comparative discussion on whether the diseases affect men and women workers in different ways could be focused, & comparative discussion on which section’s workers are more vulnerable could also give more apprehended results on the existing health problems of the RMG workers.	Strong
Rahman & Rahman, 2013 [28] (Bangladesh)	Sickness and treatment: a situation analysis among the garments workers.	Descriptive type of cross-sectional study. Quantitative. Sample size: 522. Male: 20%. Female: 80%. Study location: Dhaka. Respondents were selected using purposive sampling procedure for face to face interviews. Structured questionnaire was used. Data were analyzed manually & also using computer.	To Identify morbidity pattern, duration of illness among garment workers & to determine their treatment seeking behavior during illness.	79% respondents were suffering from illness during the last 02 months: • Female sufferers: 33.6%. • Male sufferers: 10%. Symptoms of common illness: Loose motion (38%), Cough (29%), Breathlessness (28%). Most diagnosed diseases: Diarrhea (40.54%), Common cold (22.5%), Respiratory tract infections (15.1%).	No statistical test was done. The causes of health vulnerabilities were overlooked.	Moderate
Akhter et al., 2010 [23] (Bangladesh)	Health and occupational safety for female workforce of garment industries in Bangladesh.	Quantitative. Sample size: 300 (female only). Study location: Dhaka. Data were collected from 20 factories based on questionnaire. SPSS was used to analyze the data.	The study focused on the common health problems of the respondents and also the causes of these problems.	Common health problems: Headache (54%) Back pain (54%) Allergy (48%) Asthma (39%) Upper back pain (36%) Eye problem (30%) Causes of health problems & unsafety at workplace: Lack of congenial & hygienic working atmospheres, sexual harassment, unavailability of toilet washroom facilities, lack of supply pure drinking water, unawareness of the management regarding safety issues, not enough exit doors.	Exclusion of male workers from the study was not rationalized. Data analyses were weak.	Moderate
Fitch et al., 2015 [30] (Bangladesh)	The prevalence and risk factors of post-traumatic stress disorder among workers injured in rana plaza building collapse in Bangladesh.	Quantitative. Sample size: 181. Female: 110. Male: 71. Multivariable logistic regression was used to analysis the data.	To know the prevalence and risk factors of post-traumatic stress disorder (PTSD) among the Rana Plaza survivors.	Prevalence of PTSD among the Rana Plaza survivors is: 60.2%. Injuries produced from the collapse of Rana Plaza: Fracture (42.5%), Crush/pressure injury (40.3%), Superficial (26%), Concussion (internal injury) (21.6%), Dislocation/sprain/strain (21%), Amputation (4.4%). Body parts injured: Back (51.4%), Lower extremities (47%), Face/head (24.3%), Trunk/internal organ (21.6%), Upper extremities (20.4%), Neck (7.7%), Whole body (1.7%).	Other diseases/health problems (such as trauma, psychological trauma, nervous breakdown, eye sight problem etc.), except to injuries, produced from the Rana Plaza collapse could also be focused.	Strong

### Physical health vulnerabilities

RMG workers face physical health vulnerabilities due to the workplace conditions and occupational tasks involved in their employment. Studies revealed some remarkable health issues (that workers develop specifically due to their daily work activities) including respiratory problems [4, 7, 10, 11, 23–28], skin diseases/problems [4, 6, 23, 24, 27], musculoskeletal problems [4, 27], hearing loss [24, 27], and cardiovascular diseases [29]. In addition, some frequent and common diseases are reported, including; back/joint pain [4, 10, 23–25], cough and common cold [4, 7, 10, 26, 28], headache [10, 23, 24, 26], eye problem and loss of sight [4, 23–25], hepatitis (jaundice) [4, 10, 24], fever [4, 11, 26], diarrhea [11, 26, 28], and gastric pain [4, 10]; all which result in RMG workers’ health status becoming vulnerable. Factors reported as contributing to these illnesses and diseases include poor nutritional status and imbalanced diet [4, 11], poverty [26], unhealthy workplace environment [23, 25, 27], and the components of chemical hazards such as dust, smoke, mist, fumes, and dusty raw materials [22, 24]. In addition, less nutritional intake (as the garment workers usually receive low wages from their work which minimizes their capacity of buying enough nutritional foods) as well as few and short breaks during daily routine work further contributes to poorer health and leads to being underweight, anemic, and iron deficient [11, 26].

Overall the most common health issue reported for RMG workers is respiratory problems (which includes breathing-related difficulties, cold, cough, chest tightness, asthma, nasal congestion, abnormal lung function) mainly produced from the dust of raw materials [4, 7, 11, 27, 28]. Workers are affected by inhaling dust in the workplace which results in these ongoing respiratory problems. Personal protective equipment, such as the provision of face masks, which when worn in dusty environments can reduce inhalation of irritants, were rarely reported. Furthermore, participants were reported as being reluctant to use such masks and other safety equipment due to improper fitting and the obstacles to do their work efficiently [7, 27]. Since respiratory problems directly affect the lung functions (including dyspnea, breathing difficulties, and the development of chronic lung disease), the productivity of the workers reduces [7]. Moreover, the workers might not be able to work for a longer period due to the lung dysfunctions and other respiratory symptoms. As a result, the RMG worker’s future employment opportunities can be reduced.

### Psychological health vulnerabilities

In addition to the physical health issues reported, the identified studies also showed that garment workers are vulnerable to psychological issues. The common symptoms of psychological issues identified in the literature include trauma [4, 30], work stress [31], depression and its associated risk factors such as insomnia, hypertension, and heart attack [8, 25], somatic illness, anxiety and social dysfunction [8], and sleeplessness [10]. The causes of work stress, which mainly fuels psychological vulnerabilities, are primarily related to work associated demands (i.e. long working hours, worries about mistakes, time pressure, exposure to abusive language and emotional abuse, and physical demand) [10, 31] and work-related values (i.e. lack of freedom at work, lack of job promotion prospects, part-time work and job insecurity) [10, 25, 31]. In addition, long working hours, the monotony of the type of work, work-related injury, feeling unsafe in the workplace, and lack of recognition have been identified as the major causes of psychological health vulnerabilities among the RMG workers [22].

### Health vulnerabilities among the garment workers: evidence from the mixed-method findings

Among three of the mixed methods studies that were retrieved through this literature search, two studies recruited only female workers as respondents [17, 32], while the third study recruited both female and male workers [9]. These mixed-method studies used FGD (focus group discussion) as a tool to collect the data for the qualitative part along with survey questionnaires for the quantitative component. These mixed-method studies focused on musculoskeletal related health problems of the RMG workers of Sri Lanka and India. These studies identified some common symptoms (such as pain in the knee, shoulder, neck, back, hand and wrist) of musculoskeletal problems which are related to the increased age and work tenure of the participants [17]. In addition to long work hours, sitting in a bent position for a long time and reduced general movement during work hours were also reported to contribute to the musculoskeletal problems [9]. Besides the musculoskeletal problems, these mixed-method studies also revealed other important health issues experienced by the participants which included headaches, work-related injuries and stress, and emotional abuse (see Table 2).

**Table 2:** Research on health vulnerabilities in the RMG sector: based on mixed methods

Author(s) & Year of publication (Country)	Title	Methodology/purpose	Theme/focusing point	Findings/outcomes	Limitations	Quality grading
De Silva et al. 2013 [32] (Sri Lanka)	Health status and quality of life of female garment workers in Sri Lanka	Mixed-methods. Sample size: 1058 (female only). Used structured questionnaire. Respondents were selected randomly. 4 FGD (each consists 6 respondents). Used Stata to carry out tabulations.	To understand the quality of life and the health problems of the female garment workers.	Reported health vulnerabilities issues: (a) Presence of musculoskeletal problem: 15.5% Common symptoms of musculoskeletal problem: Back pain (57.3%), Pain in knees (31.7%), Shoulder (9.1%), Neck (6.7%). (b) Headaches: 7.6% (c) Work related injury/accidents: 5.6% (d) Respiratory problems: 3.4% (e) Dermatological problem: 2.2% (f) Emotional abuse: 0.5%	Exclusion of male respondents was not explained. The causes of health vulnerabilities were not exposed in this study.	Strong
Lombardo et al., 2012 [17] (Sri Lanka)	Musculoskeletal symptoms among female garment factory workers in Sri Lanka	Cross sectional study. Mixed-methods. Sample size: 1058 (quantitative), & 24 for FGD (qualitative) (female only). 1st phase: FGD of 4 groups (each group consists of 6 female workers). 2nd phase: primary data was collected from 1058 female garment workers who were randomly selected for interviewing using a self-administered questionnaire. Descriptive statistics were calculated, bivariate & multiple logistic regression analysis was done.	To assess the presence of musculoskeletal symptoms among the respondents & to find the correlations between these symptoms and socio-demographic factors of the respondents.	Prevalence of musculoskeletal symptoms among the respondents: 16%. Common symptoms (affected body area): Back (57.3%) Knees (31.7%) Shoulders (9.1%) Hand & wrist (7.3%) Neck (6.7%) Forearm & elbow (3%) Causes of musculoskeletal symptoms: Increased age, job tenure, & work nature (i.e. long standing position while working).	Male workers were excluded.	Strong
Saha et al., 2010 [9] (India)	Health status of workers engaged in the small - scale garment industry: how healthy are they?	Cross-sectional study. Mixed-methods. Sample size: 112. Male: 86. Female: 26. Used semi-structured questionnaire. 2 FGDs (each consisted of 9 respondents) were also conducted.	To explore the relationship between morbidity profile, socio-demographic & occupational factors with musculoskeletal disorders.	Prevalence of musculoskeletal symptoms (chief complaints) among the respondents: 69.64%. Affected body area: Neck (64.10%), Low back (41.03%). Common symptoms: Pain (69.23%), Weakness (38.46%). Causes: Bad working environment, remaining in a bent position for long time, ill ventilation, poorly illuminated room. Other health problems: Malnutrition (37.50%), Acidity & heart burn (26.79%), Skin diseases (25%), Insomnia (21.43%), Hypertension (16.07), General weakness (14.29%), Problems with vision (12.05%). • Stress, especially among the female workers, is another rising & alarming problem.	Although the title of the article gives the impression to focus on the overall health status of the workers, this piece of work predominantly pointed out only musculoskeletal problems of the workers	Strong

### Variations of health vulnerabilities: specific country perspectives

The bulk of the literature has originated from studies of Bangladeshi RMG workers, with lesser research being produced from other South and Southeast Asian countries. Nevertheless, reports appear to indicate some country or local origin variability. Analysis of the identified studies indicates that workers in Bangladesh and India face almost the same kinds of health vulnerabilities, whereas RMG workers in China, Thailand, and Cambodia appear to experience different kinds of health vulnerabilities. For example, general health issues (such as back/joint pain, headache, jaundice, eye problem, fever, diarrhea, work stress, gastric pain), produced from unhygienic workplace conditions, are more frequently reported as acute among the Indian and Bangladeshi RMG workers [4, 8, 10, 22–29, 31]. On the other hand, respiratory symptoms, occupational allergic contact dermatitis, poor nutritional related health issues are more prevalent among the Chinese, Cambodian, and Thai RMG workers [6, 7, 11]. Adding additional cultural variation, it appears that RMG workers of Sri Lanka are comparatively healthier than workers from other developing countries, seemingly due to the availability of doctors in the factories, regulating welfare activities and labour rights imposed by the government, and the comparatively higher education level among the Sri Lankan RMG workers [32]. In addition to the above reported physical health vulnerabilities, the literature also emphasizes differences regarding psychological health vulnerabilities which also vary between geographic locations. For example, psychological health-related vulnerabilities are more often reported among the RMG workers in Bangladesh (*n* = 7), India (*n* = 2), and Cambodia (*n* = 1), while not reported in other countries.

The differences in terms of health vulnerabilities from country to country found in the literature signify that the RMG workers of Bangladesh and India are more vulnerable than the other workers of South and Southeast Asian countries. There was variety in the methodological designs and aims of the research retrieved through this review influencing the reported outcomes. All the included studies from Bangladesh (*n* = 10) utilised quantitative designs, including five studies identified as cross-sectional studies. These studies variously focused on the causes and consequences of physical and psychological health vulnerabilities, correlations between developing a disease and the physical setting of the factories, morbidity patterns, and workers’ health-seeking behavior. Three out of four studies from India used quantitative methods (two of them being cross-sectional studies) and one study used mixed-methods. The primary objectives of the four studies were to identify correlations between health status with workplace safety and working conditions, types of common diseases among the workers, factors affecting mental health and physical health, and the morbidity profile of the workers. The studies from Sri Lanka (*n* = 2) used mixed-methods, one of which was a cross-sectional type of study and focused on the quality of life of the workers and their health problem, especially musculoskeletal problems. One study from Thailand was identified as a cross-sectional study (quantitative) focusing on risk factors related to respiratory symptoms among the workers. Similarly, the study in China followed a quantitative method using a cross-sectional approach to investigate the causes and consequences of occupational allergic contact among RMG workers. Lastly, one study conducted among the RMG workers of Cambodia used the quantitative cross-sectional to focus on different kinds of health risks and their association with poor nutritional status. Regardless of differing designs, the reported between country differences in health vulnerabilities indicate the RMG workers of Bangladesh and India are more vulnerable than the other workers of South and Southeast Asian countries. Yet, the health vulnerabilities of Bangladeshi and Indian workers are similar.

### Summary of health hazards: the country perspective

The most common workplace hazards leading to health vulnerabilities in RMG workers in the South and Southeast Asian countries are presented in Table 3. These hazards are broadly divided into four groups: (a) ergonomic hazards; (b) physical hazards; (c) psychological hazards; (d) mechanical hazards; and (e) chemical hazards using the grouping by Padmini and Venmathi [22], as these hazards are directly linked to the health vulnerabilities of the RMG workers. Where these hazards are present, the greater the risk and vice versa in the absence of such hazards, workers are less vulnerable. For example, while not all countries are represented in the literature, Bangladeshi and Indian RMG workers experience the majority of workplace hazards reported (Table 3) among the South and Southeast Asian countries represented in the literature retrieved for this systematic review. Therefore, it appears the employment conditions and occupational tasks of these workers lead them to higher health vulnerability and harms than the other South and Southeast Asian countries (Tables 1 & 2). However, evidence from Sri Lanka shows a different profile. While culturally similar to neighboring India and Bangladesh, the health vulnerabilities of the workers are reportedly less than these neighboring countries. Thailand RMG workers fare worse. RMG factories of China and Cambodia appear to have higher workplace quality, leading to fewer vulnerabilities, where only one hazard has been reported to date (i.e. chemical hazards, leading to physical health vulnerability, in the Chinese factories, and psychological hazards in Cambodian factories). All remaining countries have multiple vulnerabilities reported.

**Table 3:** Characteristics of workplace hazards exist in the South and Southeast Asian countries

Country & References	Ergonomic hazards (related to musculoskeletal problems)	Physical hazards	Psychological hazards	Mechanical hazards	Chemical hazards
Bangladesh Khan et al., 2015 [24]; Steinisch et al., 2014 [31]; Ahmed & Raihan, 2014 [4]; Steinisch et al., 2013 [10]; Fitch et al., 2015 & 2017 [25, 30]	- Less/no break during the work -Long working hours -Lack of safety tools	-Inadequate light -Noise pollution -Inadequate ventilation -Congested & overcrowding workplace -Unavailability of separate toilets -Unhealthy workplace environment	-Lack of job promotion prospects -Job insecurity -Trauma, depression, & anxiety -Unwanted sexual contact/harassment -Long working hours, -Time pressure & worries about making mistakes -Exposure to abusive language -Lack of recognition -Inadequate payment/low income -Lack of security at workplace -Lack of psychological counseling	-Collapse of factory building (i.e. collapse of Rana Plaza in 2013), & -Fire in the factory building (i.e. fire in the Tazreen Fashion in 2012)	-Dust -Chemical dying -Chemical compound
Cambodia Makurat et al., 2016 [11]	–	–	-Low salaries (cause of poor nutritional status of the workers & mental stress of the workers) -Anemia	–	–
China Chen et al., 2017 [6]	–	–	–	–	-Dusty work materials -Dyes (These cause occupational allergic contact dermatitis)
India Padmini & Venmathi, 2012 [22]; Shanbhag & Bobby, 2012 [8]; Parimalam et al., 2007 [27]; Saha et al., 2010 [9]	-Inadequate seating & standing arrangements for the workers -Bent positioning to work -Obsolete machinery -Improper lifting or movement of heavy loads -Lack of safety tools (these cause musculoskeletal problems)	-Noise -Lighting problem Electric shock -Hot conditions inside the factory -Ventilation problems -Poorly illuminated rooms	-Monotonous type of work -Feeling of risks at workplace -Long working hours -Lack of recognition -Depression -Anxiety -Social dysfunction	-Fire hazard	-Dusts -Fumes -Mist -Smoke -Fog or smog -Smoke Liquids or gases -Solids -Vapours
Sri Lanka De Silva et al., 2013 [32]; Lombardo et al., 2012 [17]	-Increased age of the workers -Job tenure -Work nature (i.e. long standing position while working) (These cause musculoskeletal problems)	–	-Emotional abuse	–	
Thailand Chumchai et al., 2015 [7]	–	-Inadequate ventilation -Inappropriate workplace & poor workspaces	–	–	-Fabric dust (The cause of respiratory problems)

(−) indicates the absence of the category

### Quality assessment

The merit and quality of the final 19 articles were assessed using the criteria of EPHPP (Effective Public Health Practice Project) [33], and CASP (Critical Appraisal Skills Programme) [34] tools. The EPHPP tool was used to assess the quality of the quantitative studies and quantitative phase of the mixed-method studies (Part A and B of Table 4) while CASP was used to assess the quality of the qualitative phase of the mixed-method studies (Part C of Table 4).

**Table 4:** Assessing the quality of the papers

(A) Assessing the quality of the quantitative studies through using EPHPP (Effective Public Health Practice Project) tool (yes = 1, no = 0)												
Author(s) & Year of publication	Selection Bias	Study Design	Confounders	Blinding	Data Collection Methods	Withdrawals & Drop-outs	Intervention Integrity	Analyses	Scores Attained	Ratings (1–3 = weak, 4–6 = moderate, 7–8 = strong)		
Chumchai et al., 2015 [7]	1	1	1	1	1	1	1	1	8	strong		
Shanbhag & Bobby, 2012 [8]	1	1	1	1	1	1	1	1	8	strong		
Chen et al., 2017 [6]	1	1	1	1	1	1	1	1	8	strong		
Padmini & Venmathi, 2012 [22]	1	1	1	0	1	1	1	0	6	moderate		
Makurat et al., 2016 [11]	1	1	1	1	1	1	1	1	8	strong		
Parimalam et al., 2007 [27]	1	1	1	1	1	1	1	1	8	strong		
Ahmed & Raihan, 2014 [4]	1	1	1	1	1	1	1	1	8	strong		
Fatema et al., 2014 [29]	1	0	1	0	1	1	1	1	6	moderate		
Hasnain et al., 2014 [26]	1	1	1	1	1	1	1	1	8	strong		
Rahman & Rahman, 2013 [28]	1	1	1	0	1	1	1	0	6	moderate		
Steinisch et al., 2013 [10]	1	1	1	1	1	1	1	1	8	strong		
Steinisch et al., 2014 [31]	1	1	1	1	1	1	1	1	8	strong		
Khan et al., 2015 [24]	1	0	0	1	1	0	1	1	5	moderate		
Fitch et al., 2017 [25]	1	1	1	1	1	1	1	1	8	strong		
Akhter et al., 2010 [23]	0	1	0	1	1	1	1	0	5	moderate		
Fitch et al., 2015 [30]	1	1	1	1	1	1	1	1	8	strong		
(B) Assessing the quality of the quantitative part of mixed-method studies through using EPHPP (Effective Public Health Practice Project) tool (yes = 1, no = 0)												
Saha et al., 2010 [9]	1	1	1	1	1	1	1	1	8	strong		
De Silva et al., 2013 [32]	1	1	1	1	1	1	1	1	8	strong		
Lombardo et al., 2012 [17]	1	1	1	1	1	1	1	1	8	strong		
(C) Assessing the quality of the qualitative part of mixed-method studies through using CASP (Critical Appraisal Skills Programme) tool (yes = 1, no = 0)												
Author(s) & Year of publication	Clear research goal/aims	Appropriate methodology	Appropriate research design	Appropriate recruitment strategy	Justification of the way of data collection	Researcher & participants relationship considered	Consideration of ethical issues	Rigorous data analysis	Explicit findings	Value of research	Scores attained	Ratings (1–4 = weak, 5–8 = moderate, 9–10 = strong)
Saha et al., 2010 [9]	1	1	1	1	1	1	1	1	1	1	10	strong
De Silva et al., 2013 [32]	1	1	1	1	1	1	1	1	1	1	10	strong
Lombardo et al., 2012 [17]	1	1	1	1	1	1	1	1	1	1	10	strong

The quantitative articles were assessed using the criteria of the EPHPP tool which includes the following criteria: 1) selection bias; 2) study design; 3) confounders; 4) blinding; 5) data collection methods; 6) withdrawal/dropouts; 7) intervention integrity/respondent’s exposure of interest; and 8) analyses. Assessment scoring is based on each criterion carrying one point, with a total possible maximum of 8 points. As per this assessment model, articles scoring 1–3 points were considered as ‘weak’ and scored 4–6 points were regarded as ‘moderate’, with articles scoring 7–8 points considered as ‘strong’.

In addition to the above criteria for the EPHPP tool, the criteria of the CASP tool was used to assess the quality of the qualitative phase of the mixed-method papers (noting that no exclusively qualitative papers were identified in the systematic search). These criteria are: 1) clear research goal/aims; 2) appropriate methodology; 3) appropriate research design; 4) appropriate recruitment strategy; 5) justification of the way of data collection; 6) researcher and participants relationship considered; 7) consideration of ethical issues; 8) rigorous data analysis; 9) explicit findings; and 10) value of research.

In terms of assessing the quality of the qualitative part of the mixed-method studies, scores 1–4 was considered as ‘weak’, 5–8 as ‘moderate’, and 9–10 as ‘strong’ (here one mark was given for meeting one criterion out of the total of maximum 10 marks).

The quality assessment procedure represents eleven of sixteen quantitative studies as ‘strong’ and the remaining five as ‘moderate’ (Table 4A). The reported limitations of these five studies comprise: Padmini & Venmathi [22] and Rahman & Rahman [28] **-** blinding and analyses; Fatema et al. [29] **-** study design and blinding; Khan et al. [24] **-** study design, confounders, and withdrawals and Drop-outs; Akhter et al. [23] **-** selection bias, confounders, and analyses. In addition, all the mix-methods studies (*n* = 3) were rated as ‘strong’ as per Table 4 (Part B and C).

## Discussion

A total number of 19 research articles were retrieved for this systematic review, of which 16 used quantitative methods and the remaining three were mixed methods studies. The quality of the quantitative articles varied with 11 articles regarded as ‘strong’ and five assessed as ‘moderate’ after applying the criteria of the EPHPP and the CASP tools. All mixed methods articles (n = 3) were considered as ‘strong’. In a synthesis (considering the findings of the studies used in this systematic review), we demonstrate that RMG workers of South and Southeast Asia are vulnerable to different types of physical and psychological issues, which are mainly related to hazardous workplace conditions and lack of safety equipment, and occupational health and safety regulations [27]. Questions might arise whether RMG workers are unique or whether factory workers from the same geographic regions are also prone to health vulnerability in other workplaces. Research findings have demonstrated that workers in the baking industry, cotton/silk mill industry, tannery industry, and export processing industry are also predisposed to health vulnerabilities [16, 35–39]. While frontline RMG workers are shown across the literature as vulnerable, those in management positions in the RMG sector are not. For example, one study demonstrates that the managers in the RMG factories are less likely to be affected by workplace-related hazards compared to the workers of the same RMG factory [6]. Since managers are mostly involved in supervision at the workplace, therefore, it can be argued that workers are more prone to health vulnerabilities because they do all kinds of hazardous work in unhealthy working conditions. In addition to the conditions causing vulnerability, the workers have also been reported as being reluctant to undertake health-seeking behavior due to their poor economic status, low educational level [26], and general disempowerment within the setting in which they work.

The evidence from countries other than Bangladesh and India was minimal (Cambodia (*n* = 1), China (n = 1), Thailand (n = 1), and Sri Lanka (*n* = 2)), thus it remains unknown whether the RMG workers of these countries are less vulnerable to health risks, or there has not been the interest in understanding their health vulnerabilities. If they are less vulnerable then what are the reasons for these variations and what contributes to better health outcomes in these countries? For example, in Sri Lanka it appears to be the presence of medical staff employed in the factories. However, pre-existing conditions (such as better health prior to RMG factory employment), different laws and focus on occupational health in different counties, and /or the influence of multinational countries to pressure higher quality work environments, which increase the health status of the workers, may explain this difference. An alternate explanation may be that more research has been conducted in India and Bangladesh than the other countries (given the large presence of RMG factories, and high profile disasters affecting workers) [30], and thus more is generally known about the health vulnerabilities of these workers. Adding to this, it is possible that government controls over RMG sector in some countries may result in an accurate picture of health being unobtainable to researchers and thus not reflected in the academic literature. For example, there is the scarcity of occupational health risks related data in the Chinese clothing industry [6], and it is reported that the governments of China and Vietnam control over the media, internet, including control over what information to be disseminated [40]. Therefore, it is possible that the governments of these countries may not allow the academicians or researchers to explore RMG workers’ health vulnerabilities extensively through conducting empirical research. Thus, at this juncture, the reasons for this variation are unclear.

Before focusing on limitations and strengths of this systematic review, an attempt can be made to address the following questions raised by the findings of this review: first, is the health of the RMG workers’ a priority for factory owners? Second, are the reasons for poor health status-related solely to their occupation? Last, do health vulnerabilities of the RMG workers demand more research to better determine the causes and consequences of this work on the people performing these tasks?

The 19 articles used in this systematic review successfully emphasized different issues (i.e. diseases, lack of nutrition, workplace hazardous conditions) which create health vulnerabilities for RMG workers. However, factory owners’ responsibility was not clearly explained in the studies. Yet, as Kabeer and Mahmud [41] clearly pointed out, factory owners’ tendency (i.e. they tend to invest less money to ensure workers safety at the workplace) to maximize profit through minimum investment clearly contributes as a prime cause for the problems experienced by the RMG workers. Quality workplace facilities (such as cleanliness of workplace, availability of safety equipment and fire exits, regular training on safety issues, and temperature control inside the factory) might be an additional investment for the factory owners which they may not be willing to make within such an economically driven paradigm [41–44]. As a result, it appears that millions of RMG workers are bound to work in unsafe and hazardous workplace conditions which increases morbidity and mortality within this population.

As with all workers, RMG workers’ rights to work in a safe workplace are ensured through the international labor laws and national labor laws of each country. The International Labor Organization (ILO) proposes freedom to express the experience of the workplace, social security of the workers and their family members, and to treat women and men equally at the workplace [18]. Thus, ILO is in favor of safeguarding labor rights and safety related issues through proposing several conventions such as the occupational safety and health convention (1981, No. 155), the social security convention (1952, No. 102), the occupational health services convention (1985, No. 161), the employment injury benefits convention (1964, No. 121), and the promotional framework for occupational safety and health convention (2006, No. 187) [18]. Yet, the literature suggests that such regulations have not been adopted by member countries in the South and Southeast Asian regions where RMG factories are located, and thus there is little guarantee of protection of workers’ rights.

After analyzing the findings of the research articles identified for this systematic review, several solutions to reduce worker vulnerability are emphasized: (a) the provision of safety equipment to wear during work; (b) fabric dust needs to be dealt cautiously in hygienic ways; (c) availability of qualified MBBS (Bachelor of Medicine and Bachelor of Surgery) doctors in the factory premises and supplying necessary medicines free of charge; (d) regulating frequent meetings between management and workers about safety issues and also mandatory safety training before commencing employment; (e) providing clear employment contracts where minimum standard salaries are ensured; and (f) ensuring comfortable and decent working positions [4, 6, 7, 9, 11, 17, 24, 25, 27].

### Strengths and limitations

As with all reviews, there are inherent limitations. First, this study did not include research articles published before July 2007. Second, this review only included literature published in English which was a practical consideration due to author’s language limitations as well as to capture high quality, peer-reviewed literature. Third, working papers, reports or other grey literature sources, books and book chapters which may be published on the basis of primary data were not included. Thus, there is a possibility that these sources may have retrieved additional results. Fourth, the majority of the studies predominantly focused on the female RMG workers’ health vulnerabilities. Therefore, the findings of this review may be similar, or different, for male workers. It is also not clear whether male workers are less vulnerable to health vulnerabilities or the inclusion of female workers is solely due to the higher proportion of women employed in this sector. Fifth, sudden factory disasters are common in the countries of South and Southeast Asia affecting RMG workers [30]. The impact of a sudden factory disaster on physical and psychological health remains almost entirely unknown [42], and was only identified as a vulnerability in one of the located studies [30]. Lastly, this systematic review failed to uncover any research covering both physical and psychological components, nor was any purely qualitative research located.

The above-mentioned limitations were mitigated by several strengths. First, as the RMG sector is relatively new, it is likely that the majority of research is quite recent. Second, while we limited our search to English only, we retrieved publications from the included countries, thus demonstrating that at least some academics and researchers have been able to publish their research in English-language journals even though English is not their first language, extending the debate about this important topic beyond the local discussion. Third, the databases which were searched have a broad scope and thus there it is likely that relevant literature was not bypassed. Fourth, the majority of the studies focused on female respondents. This is reflected in the workforce in almost all of the regions from which these studies are reported are also predominantly female. Fifth, this systematic review focused solely on the specific health vulnerabilities (both physical and psychological health vulnerabilities) that the RMG workers face and also exposed the causes of these health vulnerabilities. In addition, health vulnerabilities from different country perspectives were explained so that garment workers’ health vulnerabilities of one country can be compared with another country. Lastly, it can be argued (on the basis of the searched databases) that no other systematic review has been undertaken exploring RMG worker’s health vulnerabilities in South and Southeast Asian countries. Therefore, the limitations of this paper might encourage the future researchers to conduct more work in this field particularly in those countries not yet represented in the international literature and, extending searches to other industries (such as textiles) and vulnerabilities (such as home versus work) related to the nature of the factory work they are involved in.

### Future directions

Since the majority of the research (*n* = 16) to date has used a quantitative approach, this systematic review suggests conducting more research including both physical and psychological health vulnerabilities using a mixed-method or qualitative only approach. In addition, only one study was conducted focusing on the health vulnerabilities produced from a sudden disaster [30]; yet sudden disaster is not uncommon. Therefore, more research should be conducted to explore how a sudden disaster, such as the collapse of Rana Plaza in Bangladesh, creates distinctive health vulnerabilities including psychological trauma for RMG workers. Lastly, four of the ten studies that focused on the health vulnerabilities of the RMG workers of Bangladesh were considered as ‘moderate’ quality making it difficult to accept the outcomes. Therefore, this field requires more quality research in Bangladesh context in future, given the proliferation of this sector in that country over the past two decades.

## Conclusions

The results of this systematic review suggest that RMG workers of South and Southeast Asian countries are vulnerable to several health challenges which include both physical and psychological issues. The results further suggest that many of the physical health issues are produced broadly from the nature of the work they undertake in their employment, including the unhygienic and unsafe working environments, hazardous conditions of the factories, and lack/unavailability of safety equipment. In addition, RMG workers are vulnerable to psychological vulnerabilities due to excessive workload, low wages, abusive language, job insecurity, and feeling unsafe in the workplace. In spite of this evidence, robust research has not been undertaken to illustrate the nature and extent of the problem and how it relates to other key sources of disadvantages for the RMG workers in these regions. As such, little is known about the full extent of the vulnerabilities faced by RMG workers in these regions. The Bangladeshi RMG workers in particular, face unique problems because their vulnerabilities are highly mediated by their physical and psychological health, yet more is known about the Bangladeshi workforce than other countries. Thus, the results of this systematic review are significant as they highlight the paucity of literature on the vulnerabilities of the RMG workers elsewhere in the South and Southeast Asian regions.

## Abbreviations

AIDS: Acquired immunodeficiency virus
CASP: Critical appraisal skills programme
CVDs: Cardiovascular diseases
EPHPP: Effective public health practice project
FGD: Focus group discussion
HIV: Human immunodeficiency virus
ILO: International labour organization
MBBS: Bachelor of medicine and bachelor of surgery
PRISMA: Preferred reporting items for systematic reviews and meta-analyses
RMG: Readymade garment
